# Electrospinning Synthesis of Nano-Scale MgO Fibers and Their Methylene Blue Adsorption Efficiency

**DOI:** 10.3390/ijms26051907

**Published:** 2025-02-23

**Authors:** Chunyang Ma, Hongxin He, Huaxing Li, Mengyu Cao, Fafeng Xia

**Affiliations:** 1 College of Engineering, Northeast Agricultural University, Harbin 150030, China; cyma@neau.edu.cn (C.M.);; 2 College of Engineering, Northeast Petroleum University, Daqing 163318, China; huaxingli84@gmail.com (H.L.);

**Keywords:** water pollution, electrospinning, MgO fibers, adsorption

## Abstract

Water pollution from industrial dyes like methylene blue poses severe environmental and health risks, necessitating effective wastewater treatment methods. Among various adsorbents, MgO stands out due to its high surface area, tunable porosity, and superior adsorption capabilities. This research presents the preparation of nano-scale magnesium oxide (MgO) fibers using electrospinning, followed by calcination at temperatures of 300 °C, 400 °C, 500 °C, 600 °C, and 700 °C. The effects of calcination temperatures on MgO’s surface characteristics, microstructure, crystalline phases, and adsorption performance were investigated. SEM and TEM analyses revealed that fibers calcined at 500 °C possessed the most distinct porous structure, with a coarse surface and substantial pores, which enhanced adsorption properties. XRD analysis confirmed that the 500 °C calcined MgO fibers had the highest crystallinity, particularly the (200) crystal plane. Notably, BET surface area analysis confirmed the superior adsorption properties of these fibers, making them highly effective for wastewater treatment applications. Adsorption tests for methylene blue (MB) indicated that these fibers achieved a maximum dye removal efficiency of 52.52% and an adsorption capacity of 43.11 mg/g within 90 min. The adsorption process aligned with a quasi-second-order kinetic model (R^2^ = 0.9846) and fit the Langmuir isotherm (R^2^ = 0.991), indicating monolayer chemisorption. This study underscores the effectiveness of MgO fibers calcined at 500 °C, demonstrating enhanced adsorption characteristics that are beneficial for wastewater treatment applications.

## 1. Introduction

Water scarcity and pollution are pressing global challenges that threaten human health and environmental sustainability [1,2,3]. Industrial effluents containing hazardous dyes, such as methylene blue (MB), contribute significantly to water contamination [4,5]. MB is widely used in textile, paper, and plastic industries, and its presence in wastewater is problematic due to its mutagenic effects, reduction of water transparency, and harm to aquatic organisms [6,7,8]. Therefore, the effective removal of MB from wastewater is crucial before discharge into natural water bodies.

Various treatment methods have been developed to address dye pollution, including sedimentation by flocculation, electrochemical methods, chemical oxidation, photocatalytic degradation, and adsorption [9,10,11]. Yu et al. [12] developed a reactive electrochemical Ti_4_O_7_ film using an electrochemical method to treat refractory organic matter in wastewater. The results demonstrated a COD removal efficiency of 76.2% under optimal conditions, with an energy consumption of 110.5 kWh/kg COD. Hariharalakshmanan et al. [13] employed ZnO for the photocatalytic degradation of methylene blue (MB). They found that a 25 mL suspension of ZnO nanostructures, with an MB concentration of 1 ppm and a pH of 11, achieved the highest average photocatalytic degradation. Binkadem et al. [14] investigated the adsorption properties of graphene oxide (GO) and its effectiveness in removing various harmful pollutants from industrial wastewater. The results indicated that the adsorption efficiency of MB and phosphate ions (PO_4_^3−^) was enhanced up to 4–5 times using this method. Among these, adsorption is notable for its high efficiency, cost-effectiveness, and ability to remove difficult-to-degrade pollutants. Gonzalez et al. [15] used electrospinning to prepare novel composite multifunctional nanofibers (NFs). Liu et al. [16] developed ZnO-PLLA/PLLA fiber films for air filtration via electrospinning to address the rise in environmental pollution from disposable masks. Liu et al. [17] also produced a flexible alumina–silicon composite nanofiber felt (Al_2_SiO_5_) with high-temperature resistance using electrospinning technology. A diverse range of adsorbents have been explored for MB removal, such as activated carbon, graphene oxide (GO), metal oxides, and nanofibers [18,19,20]. For instance, Kamaran et al. [21] employed asphalt-derived activated carbon (PAC) particles for automotive carbon canisters and explored the adsorption performance of butane on this material under different activation times. The research findings indicate that with the increase in steam activation time, the specific surface area and total pore volume of PAC rose by 650–1950 m^2^/g and 0.27–1.02 cm^3^/g respectively. The mesopore ratio of PAC increased along with an increase in activation time, reaching as high as 28.4% at 190 h. Wang et al. [22], in response to the diseases triggered by the long-term consumption of groundwater with high iodine content, combined Fe-Mn binary oxide with bismuth oxide and probed the adsorption performance of the composite oxide for iodide. The research outcomes demonstrate that when the dosage of the oxide was 1.0 g/L, the concentration of iodide (I^−^) was 120 μg/L, and the reaction time was 60 h, the removal rates of I^−^ and iodate (IO^3−^) in groundwater reached 53.82% and 84.74%, respectively. However, some adsorbents face limitations such as high cost, low adsorption capacity, or challenging regeneration processes.

Magnesium oxide (MgO) has attracted attention as a promising adsorbent due to its large surface area, porosity, and high adsorption capacity for organic pollutants [23,24,25]. Previous studies have reported the synthesis of MgO nanoparticles and their application in wastewater treatment [26,27]. For example, Liang et al. [28] synthesized MgO nanoparticles using a sol–gel method and demonstrated their effectiveness in removing MB from aqueous solutions. However, conventional synthesis methods often result in agglomerated particles with a limited surface area, reducing adsorption efficiency. Zhang et al. [29] successfully synthesized MgO nanomaterials by means of ultrasonic electrodeposition for the removal of Pb(II) in soil and explored the adsorption capacity and adsorption mechanism of this nanomaterial prepared under different ultrasonic powers. The results indicate that the maximum adsorption capacity of the MgO nanomaterials prepared at an ultrasonic power of 150 W was observed to be 43.7 mg·g^−1^. Li et al. [30], in an effort to counter the hazards of excessive fluoride to bones and teeth, prepared MgO-modified sucrose-derived porous carbon composite materials (MPCC) for fluoride removal. The research content revealed that the maximum fluoride adsorption capacity of MPCC at 25 degrees Celsius was 26.6 mg·g^−1^. The effects of common anions such as Cl^-^, SO_4_^2−^, and NO^3−^ on fluoride removal were negligible, while the negative influence of coexisting HCO_3_ was observed.

Electrospinning is a versatile and efficient technique for fabricating nano-sized fibers with high surface area-to-volume ratios, controlled fiber diameters, and tunable porous structures. This method is particularly significant for the preparation of nanofibers because it enables precise control over fiber morphology and diameter, which are critical for optimizing adsorption properties. Electrospinning allowed for the uniform distribution of magnesium oxide precursors in a fibrous form, facilitating subsequent calcination to develop the desired porous structure. Moreover, the process offers scalability and reproducibility, making it highly suitable for producing nanofibers for industrial applications such as wastewater treatment. The combination of electrospinning and calcination provided MgO fibers with enhanced adsorption performance, highlighting the effectiveness of this method for preparing functional nanomaterials. Alonso et al. [31] sought to address the issue of the low degradability of fossil-based plastics by fabricating nanofiber membranes via the electrospinning process to enhance the value of rice bran. Zhang et al. [32] fabricated hierarchical zeolite (ZC) by means of electrospinning technology and tested the adsorption ability of methyl blue in conventional zeolites and this material. The results indicate that ZC demonstrated a faster and higher MB removal performance compared with traditional molecular sieves. The adsorption rate constant and the maximum adsorption capacity of ZC were 20 times and 1.2 times those of conventional zeolite (ZA), respectively.

This study aims to investigate the adsorption performance of MgO nanofibers for MB removal, focusing on the influence of calcination temperature and adsorption conditions. The novelty of this work lies in the combination of electrospinning and optimized calcination conditions to produce MgO with superior adsorption capabilities. Our findings provide insights into the adsorption mechanism and highlight the advantages of MgO nanofibers over other substrates in terms of efficiency and applicability in real-world wastewater treatment. This work builds on previous research by addressing gaps in adsorption efficiency and exploring a more effective method for preparing MgO nanofibers tailored for dye removal.

## 2. Results and Discussion

### 2.1. Surface Morphology of Nano-Sized MgO

**Figure 1:**
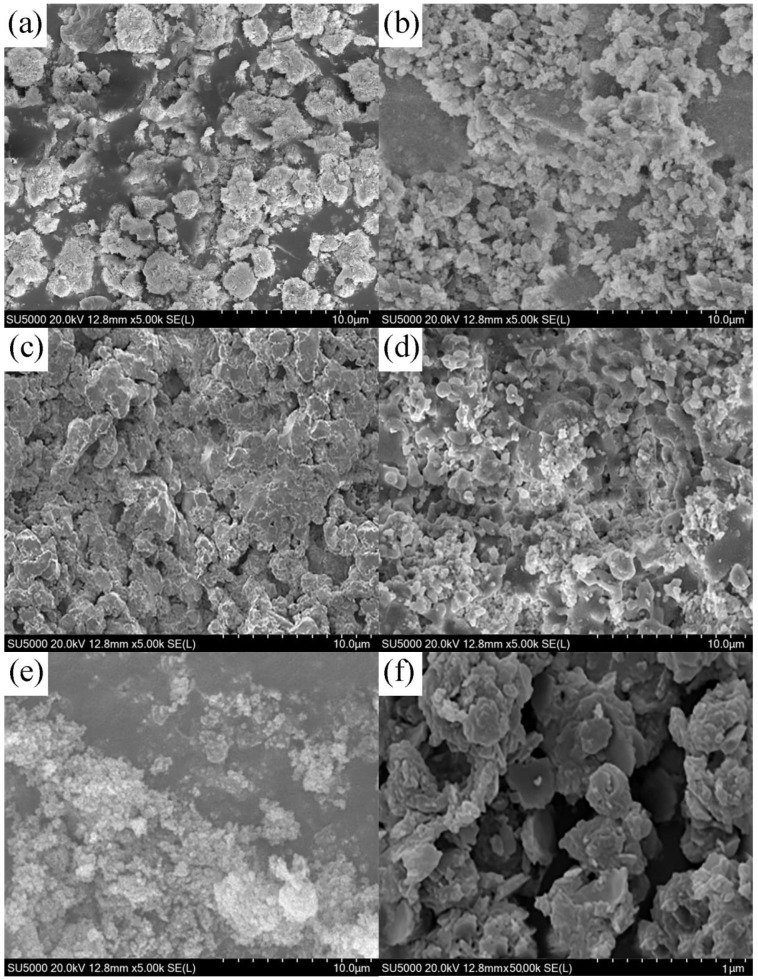
SEM images of nano-sized MgO at different target temperatures: (**a**) 300 °C, (**b**) 400 °C, (**c**) 500 °C, (**d**) 600 °C, and (**e**) 700 °C, and (**f**) magnified image of 500 °C.

The SEM images demonstrate the morphological changes in the nano-sized MgO fibers with increasing calcination temperatures. Figure 1 shows the surface morphology of the MgO fibers at different temperatures. At 300 °C (Figure 1a), the fibers displayed an irregular, aggregated structure with a rough, uneven surface. When the temperature was raised to 400 °C (Figure 1b), the particle size decreased and the porosity increased, leading to a more defined structure. At 500 °C (Figure 1c), the MgO fibers displayed the most well-developed and porous structure, with a rough surface and large pores. At 600 °C (Figure 1d), the fibers began to lose some porosity and the surface became smoother. By 700 °C (Figure 1e), the fibers were denser and more compact, with significantly reduced porosity and a smoother surface. Figure 1f provides a magnified view of the fibers calcined at 500 °C, emphasizing their highly porous and optimized morphology. This temperature produced the most well-defined porous structure. The 400 °C sample also exhibited good porosity, while the 300 °C fibers, though less developed, retained some irregular roughness. At 600 °C, the fibers started to collapse, becoming smoother and less porous. By 700 °C, the sintering effect became more pronounced, causing the fibers to become dense and compact with minimal porosity. These morphological changes are attributed to the thermal decomposition of the precursor and sintering effects, which reduce the surface area and pore structure beyond the optimal calcination temperature of 500 °C [33].

### 2.2. Microstructure Analysis of Nano-Sized MgO

**Figure 2:**
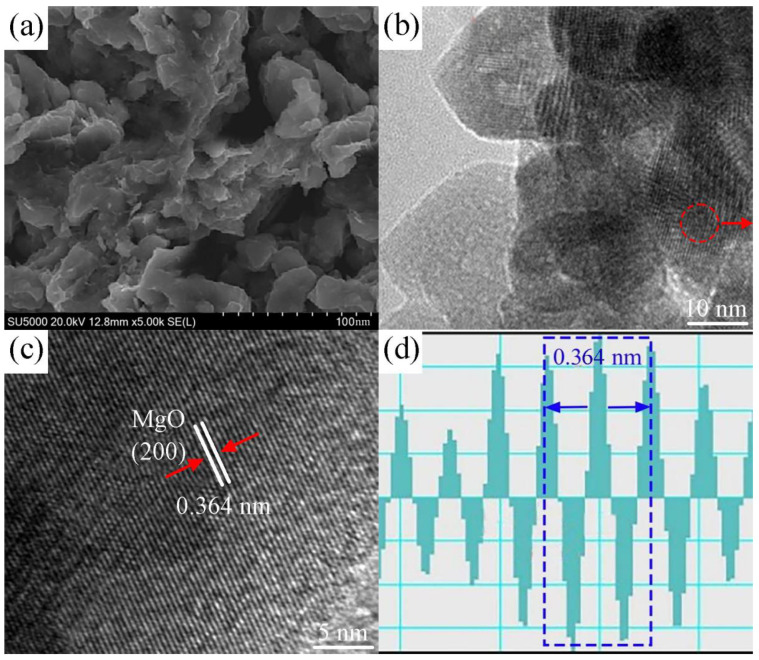
(**a**) SEM image of nano-sized MgO calcined at 500 °C, (**b**) TEM image illustrating the internal structure of the MgO particles, (**c**) HRTEM image showing lattice fringes, and (**d**) lattice spacing measurement.

Figure 2 illustrates the microstructure of the nano-sized MgO calcined at 500 °C. Figure 2a shows an SEM image of the MgO particles with a rough and porous surface morphology. Figure 2b presents a TEM image revealing the internal structure of the particles, including nanoscale crystalline domains. Figure 2c shows a high-resolution TEM (HRTEM) image, highlighting clear lattice fringes with an interplanar spacing of 0.364 nm, corresponding to the (200) plane of MgO. Figure 2d provides a detailed analysis of the lattice spacing, confirming the 0.364 nm distance between the fringes. The TEM and HRTEM images reveal well-resolved lattice fringes, indicating high crystallinity [34]. The lattice spacing of 0.364 nm corresponds to the (200) plane of MgO, confirming successful crystalline formation. The combination of porosity and crystallinity at this temperature is crucial for optimizing the material’s adsorptive and catalytic properties.

### 2.3. Phase Composition of Nano-Sized MgO

**Figure 3:**
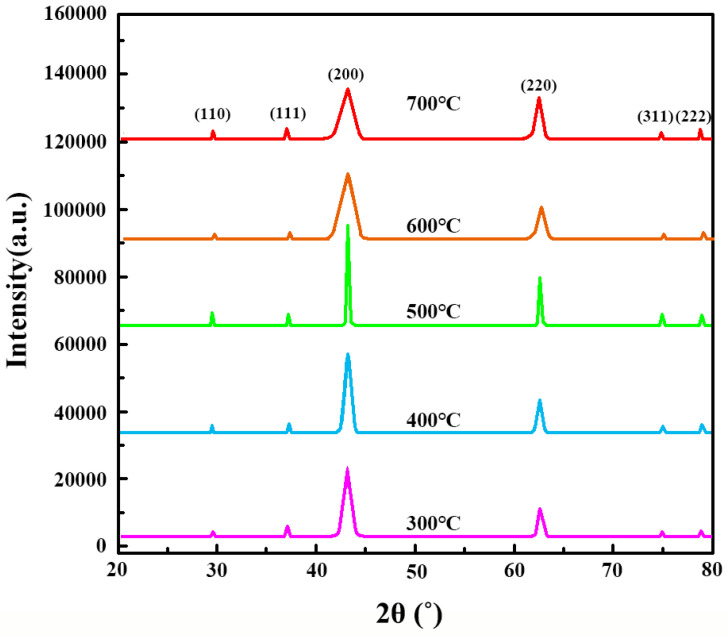
XRD patterns of nano-sized MgO calcined at different temperatures.

The XRD patterns (Figure 3) of MgO calcined at 300 °C, 400 °C, 500 °C, 600 °C, and 700 °C reveal diffraction peaks corresponding to the (110), (111), (200), (220), (311), and (222) crystal planes of MgO [35]. While the sample calcined at 500 °C showed distinct diffraction peaks, the relative intensities across the samples indicate that all the calcined materials share the same phase structure. To evaluate crystallinity more reliably, peak broadening was analyzed using the Scherrer equation, focusing on the (200) peak as a representative reflection. A zoomed-in view of the (200) peak is provided in Figure 3, revealing broader peaks for the 500 °C sample compared to those calcined at higher temperatures, suggesting smaller crystallite sizes. The calculated crystallite sizes based on the (200) peak are summarized in Table 1. These results demonstrate that the 500 °C calcined sample exhibits relatively smaller crystallite sizes, contributing to its optimized porosity and adsorption properties. Lower calcination temperatures (300 °C and 400 °C) resulted in incomplete phase formation, whereas higher temperatures (600 °C and 700 °C) led to sintering effects, reducing the surface area and structural integrity. This comprehensive analysis aligns with the SEM and TEM observations, confirming that 500 °C is the optimal calcination temperature for achieving a balance between crystallinity and porosity.

### 2.4. Adsorption Capability of Nano-Sized MgO

**Figure 4:**
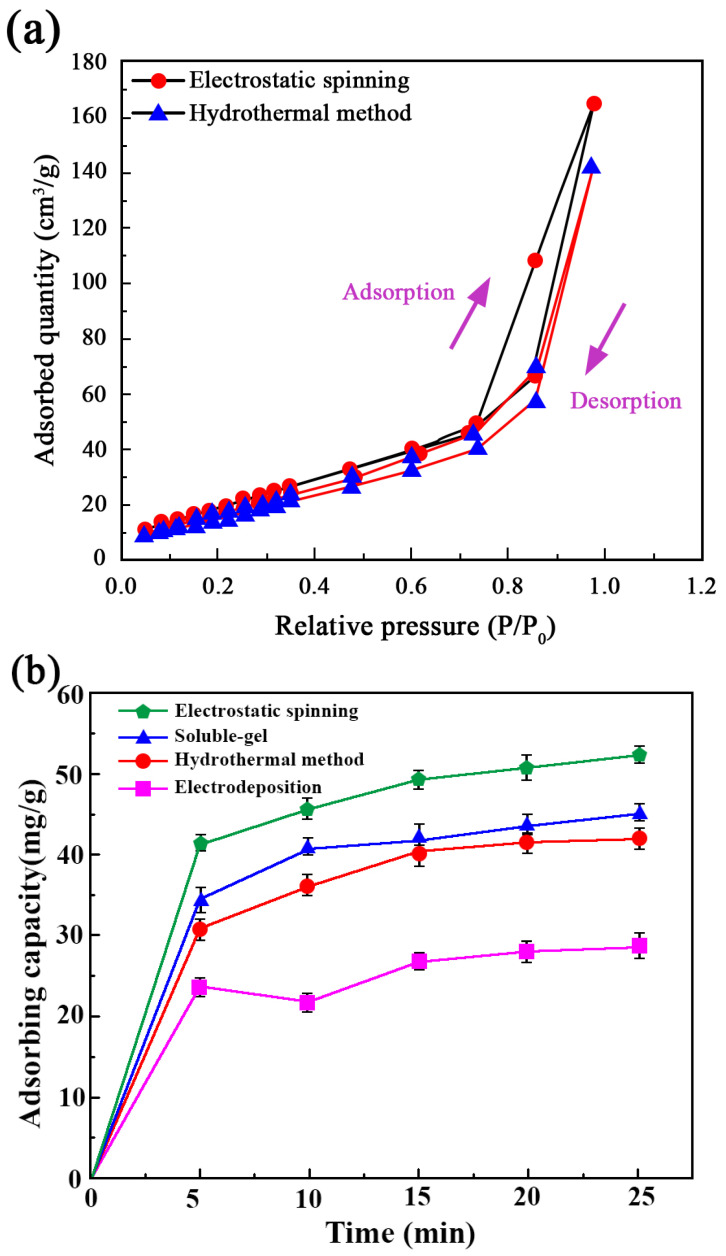
(**a**) Nitrogen adsorption–desorption isotherms of MgO prepared by different methods, including electrospinning and hydrothermal synthesis. (**b**) Adsorption capacity of methylene blue over time for MgO synthesized via different methods.

To further evaluate the adsorption capability of nano-sized MgO fibers, nitrogen adsorption–desorption isotherms were obtained using BET analysis. Figure 4a presents the nitrogen adsorption–desorption isotherms for MgO synthesized by two different methods: electrospinning and hydrothermal synthesis. The isotherms exhibit a characteristic Type IV behavior with an H3 hysteresis loop, indicative of mesoporous materials. This suggests the presence of slit-shaped pores and a well-developed porous structure, which is crucial for effective adsorption performance.

Notably, the MgO fibers prepared by the electrospinning method exhibit a higher nitrogen adsorption capacity compared to those synthesized via the hydrothermal method. The maximum adsorbed quantity for electrospinning-derived MgO reaches approximately 163.8 cm^3^/g, while the hydrothermally synthesized MgO achieves around 142.3 cm^3^/g. This difference suggests that the electrospinning-derived MgO possesses a larger surface area and higher porosity, potentially due to its finer fiber structure and enhanced pore formation during synthesis. These results further support the conclusion that nano-sized MgO fibers, especially those prepared via electrospinning, exhibit superior adsorption performance. The increased adsorption capacity highlights their potential for applications in wastewater treatment, where a high surface area and porosity are essential for effective pollutant removal. Furthermore, the adsorption kinetics experiments (Figure 4b) show that the electrospinning-derived MgO fibers achieved the highest adsorption capacity for methylene blue, reaching approximately 55.6 mg/g within 25 min. The adsorption rate was also the fastest among all tested methods, with a significant increase within the first 5 min. In contrast, MgO prepared via sol–gel and hydrothermal methods exhibits moderate adsorption capacities of around 45.7 mg/g and 40 mg/g, respectively, while electrodeposited MgO has the lowest adsorption capacity (~25.8 mg/g). This trend indicates that the higher surface area and enhanced porosity of electrospinning-derived MgO contribute to its superior adsorption performance. These results further support the conclusion that nano-sized MgO fibers, especially those prepared via electrospinning, exhibit superior adsorption performance.

### 2.5. Influence of Calcination Temperature on Adsorption Properties

A standard MB solution of 100 mg/L was proportionally diluted to prepare a series of standard solutions. The linear equation of the standard MB curve was determined to be y = 0.0024 + 0.1206x, with a high linear correlation coefficient of R^2^ = 0.99929.

**Figure 5:**
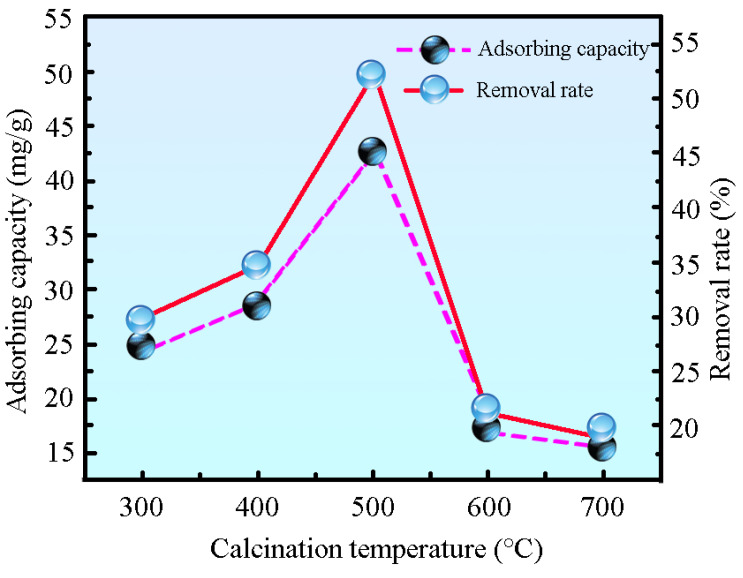
Effect of calcination temperature on adsorption properties.

After curing the precursor fibers at 200 °C for 2 h, they were calcined at 300 °C, 400 °C, 500 °C, 600 °C, and 700 °C for 1.5 h, resulting in nano-sized MgO fibers labeled accordingly. A 10 mg sample from each group was placed into centrifuge tubes containing 30 mL of 30 mg/L MB solution. The tubes were shaken at 260 r/min at room temperature for 3 h, followed by centrifugation to measure the absorbance of the supernatant. The results are shown in Figure 5. The adsorption performance increased with calcination temperature, reaching a maximum of 500 °C, with a removal rate of 52.52% and an adsorption capacity of 43.11 mg/g. Beyond this temperature, the efficiency declined due to the collapse of the fiber’s pore structure caused by excessive heat. This was due to the complete thermal decomposition of magnesium acetate tetrahydrate to MgO at 500 °C [36]. Thus, 500 °C was identified as the optimal temperature for producing MgO fibers with the highest adsorption capacity. For subsequent experiments, the fibers were cured at 200 °C and calcined at 500 °C for 1.5 h.

### 2.6. Influence of Adsorption Temperature and Time on Adsorption Properties

**Figure 6:**
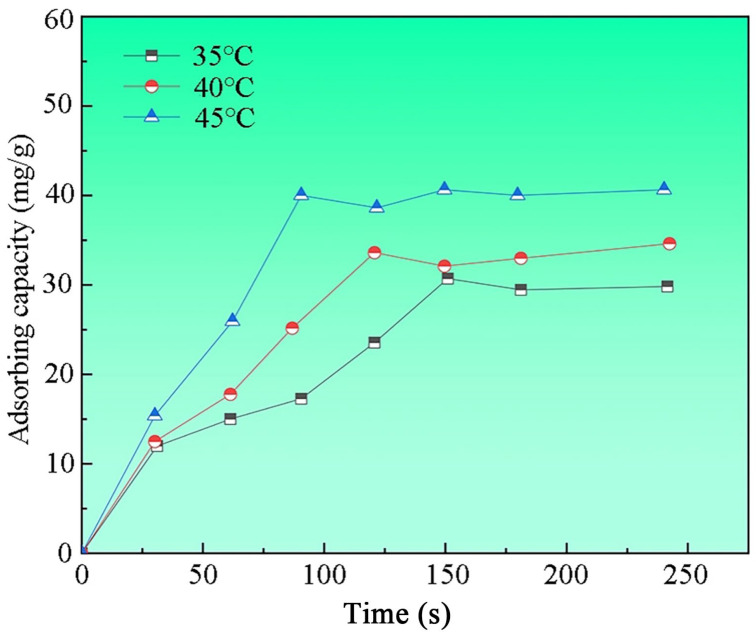
Effect of temperature on adsorption efficiency.

A 1000 mL solution of 30 mg/L MB was prepared, and 30 mL of this solution was transferred into six centrifuge tubes. Each tube was treated with 10 mg of nano-sized MgO fibers and oscillated at 35 °C and 260 r/min for 3 h. After reaching the designated time, the solution was centrifuged at 3600 r/min for 5 min. The absorbance before and after oscillation was measured using a UV-VIS spectrophotometer to calculate the removal rate [37]. Adsorption experiments at 40 °C and 45 °C followed the same procedure, except for the temperature setting. The removal efficiency results are presented in Figure 6. At 35 °C, the adsorption efficiency increased proportionally with time in the initial stage, reaching its peak at 150 min, after which equilibrium was established. At 40 °C, the efficiency also increased over time, with the maximum adsorption and equilibrium occurring at 120 min. At 45 °C, equilibrium was achieved more quickly, within 90 min, with the adsorption capacity nearing 40 mg/g. The overall analysis indicates that higher temperatures accelerate the adsorption process, leading to quicker equilibrium and enhanced MB removal efficiency.

### 2.7. Adsorption Isotherm

The adsorption isotherm represents the relationship between temperature, concentration, and adsorption capacity. To describe the adsorption behavior of methylene blue on the surface of magnesium oxide fibers, the experimental data were fitted using both the Langmuir and Freundlich isotherm models. The Langmuir isotherm equation is represented by Equation (3), while the Freundlich isotherm equation is shown in Equation (4):

(1) $$\frac{C_{e}}{q_{e}}=\frac{C_{e}}{q_{max}}+\frac{1}{q_{max}K_{L}}$$

(2) $${\mathrm{lnq}}_{e}={\mathrm{lnK}}_{F}+\frac{1}{n}{\mathrm{lnC}}_{e}$$

where *q_max_* is the maximum adsorption capacity(mg/g), *K_L_* is the Langmuir constant, K_F_ is the Freundlich constant, and n is the adsorption constant.

**Figure 7:**
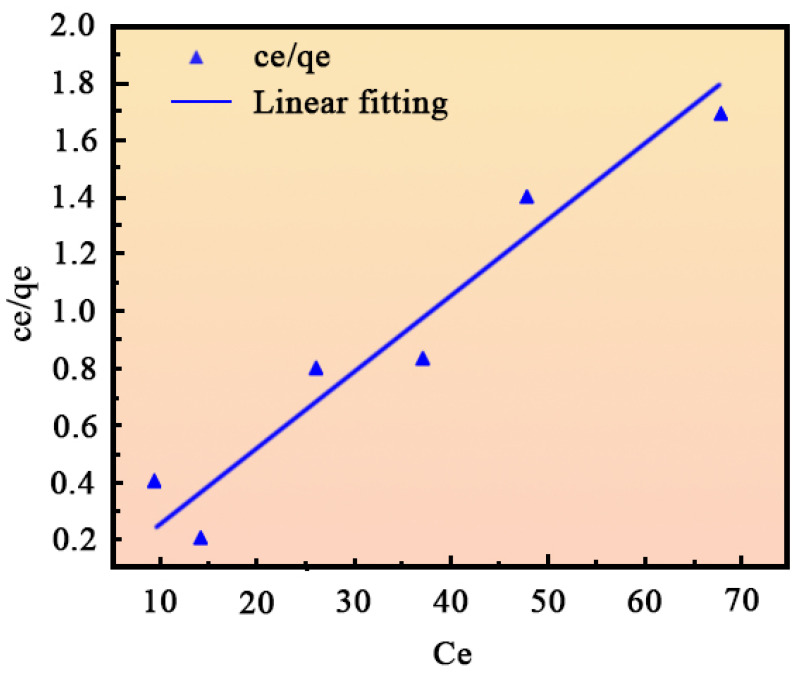
Langmuir isotherm fitting curve.

**Figure 8:**
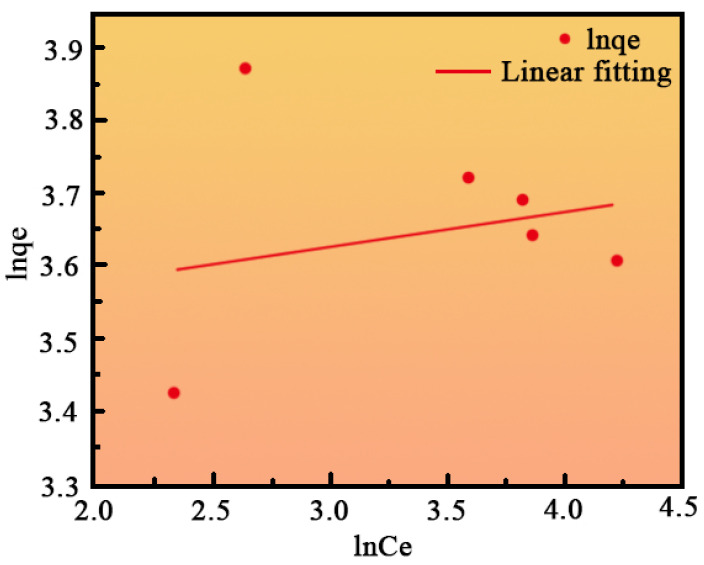
Freundlich isotherm fits curve.

Figure 7 and Figure 8 display the fitting results for the Langmuir and Freundlich isotherms, respectively, with the corresponding adsorption parameters provided in Table 1. The Langmuir model, with an R^2^ value of 0.991, offers a significantly better fit compared to the Freundlich model, which has an R^2^ value of 0.03051. This suggests that the Langmuir model more accurately describes the adsorption of MB by nano-sized MgO. Additionally, the value of R_L_ < 1 indicates that the adsorption process is favorable and likely corresponds to monolayer adsorption [38].

### 2.8. Adsorption Kinetics

Fitting the kinetic model to the adsorption data provides theoretical insights into the adsorption mechanism. The equations for the quasi-first-order and quasi-second-order kinetic models are as follows [39]:

(3) $$\ln\left(q_{e}-q_{t}\right)=\ln q_{e}-k_{1}t$$

(4) $$\frac{t}{q_{e}}=\frac{1}{k_{2}q_{e}^{2}}+\frac{1}{q_{e}}t$$

where *q_t_* is the adsorption amount at the adsorption time *t* (mg/g), *t* is the adsorption time (min), *k*_1_ is the primary kinetic adsorption constant (min^−1^), and *k*_2_ is the primary kinetic adsorption constant (g/mg·min^−1^).

**Figure 9:**
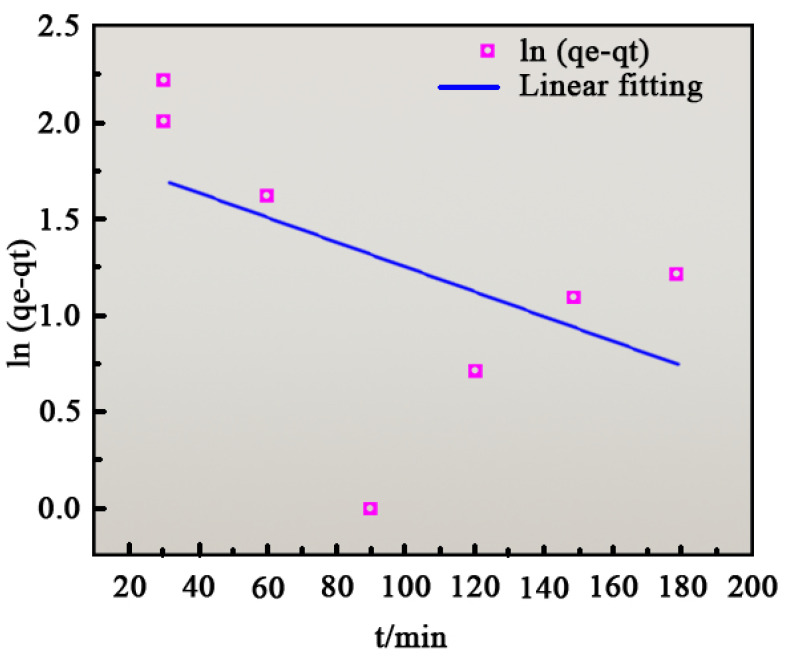
Quasi-first-order kinetic fitting curve.

**Figure 10:**
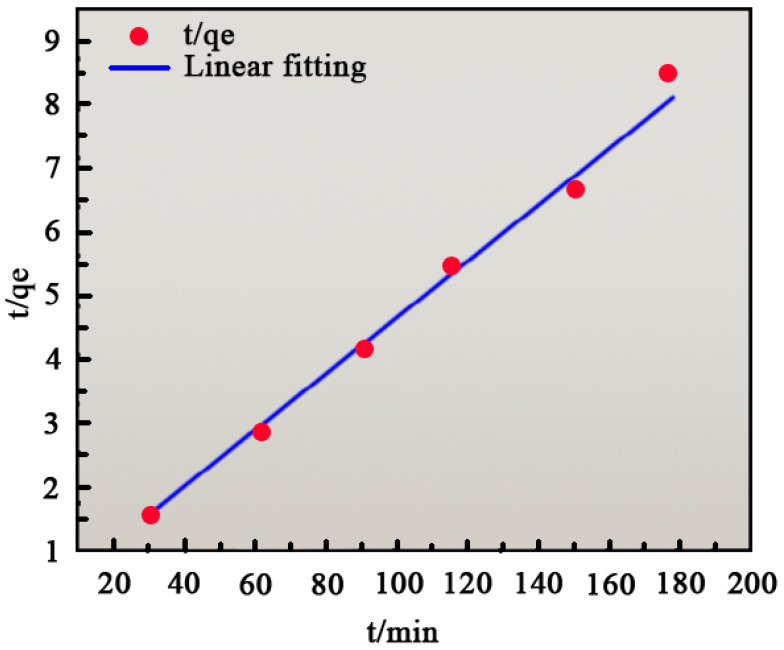
Quasi-second-order kinetic fitting curve.

Equations (5) and (6) were used to fit the experimental data. Figure 9 depicts the quasi-first-order kinetic model fit, while Figure 10 shows the quasi-second-order kinetic model fit. Table 2 presents the fitting parameters for both models. From Figure 9 and Table 3, the quasi-second-order model (R^2^ = 0.9846) exhibited a significantly higher correlation coefficient than the quasi-first-order model (R^2^ = 0.24358). This indicates that the adsorption process is better described by the quasi-second-order model, suggesting that methylene blue adsorption on MgO fibers is primarily driven by chemisorption.

## 3. Materials and Methods

### 3.1. Instruments and Reagents

The equipment used in this study include a ZNCL-TSI 100 g Magnetic Agitator from Gongyi Yuhua Instrument Co., Ltd., Zhengzhou, China; HZ-10 Huizhi Electrospinning Machine from Qingdao Nuokang Environmental Protection Technology Co., Ltd., Qingdao, China; DW-P303-1ACD1 High Voltage DC Power Supply from Dongwen High Voltage Power Supply Co., Ltd., Tianjin, China; TM-0610 Muffle Furnace from Beijing Yingan Meicheng Scientific Instrument Co., Ltd., Beijing, China; ZD-85 Constanct Temperature Oscillator from Changzhou Guohua Electric Appliance Co., Ltd., Changzhou, China; TD25-WS Centrifuge from Hunan Xiangyi Laboratory Instrument Development Co., Ltd., Changsha, China; and 721 UV-Visible Spectrophotometer from Shanghai Yidian Analytical Instrument Co., Ltd, Shanghai, China. The experimental reagents and solvents were commercially obtained as analytical-grade reagents, without any further purification.

### 3.2. Preparation of Nano-Sized MgO

**Figure 11:**
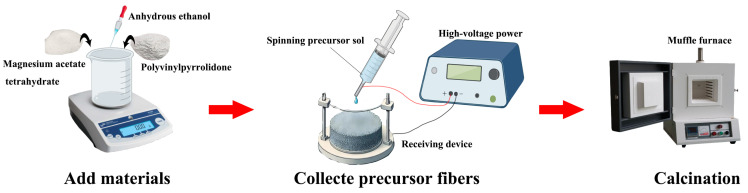
Schematic diagram of the electrospinning process for the preparation of nano-sized magnesium oxide (MgO).

Magnesium acetate tetrahydrate (2.00 g), polyvinylpyrrolidone (PVP, 0.30 g), and anhydrous ethanol (97.70 g) were mixed at room temperature for 12 h to obtain 100 g of a homogeneous spinning precursor sol. The syringe needle was connected to the positive electrode of a high-voltage power supply, and the receiving device to the negative electrode. The injection pump rate was set to 1.2 mL/h, the curing distance to 10 cm, the high-voltage parameter to 22 kV, and the drum rotation speed to 280 r/min. The room temperature was maintained at 27 °C with 30% humidity. Once uniform droplet overflow occurred at the spinning needle, the reciprocating platform was activated and spinning was initiated. The precursor fibers were collected from the electrospinning machine and placed in a muffle furnace for calcination. The temperature was raised to 200 °C at a rate of 5 °C/min and held for 2 h, and then increased to 300, 400, 500, 600, and 700 °C at 5 °C/min, with a final calcination time of 1.5 h at each temperature. The electrospinning setup is shown in Figure 11. The formulas used to calculate the removal rate (R) and equilibrium adsorption capacity (*q_e_*) are as follows:

(5) $$R=\frac{C_{0}-C_{e}}{C_{0}}\times 100\%$$

(6) $$q_{e}=\frac{\left(C_{0}-C_{e}\right)}{m}V$$

where *C*_0_ is the initial concentration of MB solution (mg/L), *C_e_* is the concentration of MB solution after adsorption (mg/L), *V* is the volume of MB (L), and *m* is the mass of nano-sized MgO fiber (g).

## 4. Conclusions

In this study, nano-sized MgO fibers were successfully synthesized using electrospinning followed by calcination at various temperatures. The calcination temperature significantly influenced the fibers’ surface morphology, crystallinity, and adsorption properties. Among the temperatures tested, 500 °C was identified as optimal, producing fibers with the most well-defined porous structure and highest crystallinity. At this temperature, the fibers demonstrated excellent adsorption performance, achieving a removal rate of 52.52% and an adsorption capacity of 43.11 mg/g for methylene blue (MB) solution. Kinetic analysis indicated that the adsorption followed a quasi-second-order model, and the Langmuir isotherm fitting suggested monolayer chemisorption. Furthermore, the BET surface area analysis confirmed the high porosity and surface area of the fibers (163.8 cm^3^/g), which contribute to their enhanced adsorption capabilities. These findings highlight that MgO fibers calcined at 500 °C offer ideal structural and functional properties, making them highly effective for environmental applications, especially in the adsorption of organic pollutants from wastewater.

## Figures and Tables

**Table 1:** The crystallite sizes for each calcination temperature were estimated using the Scherrer formula.

Temperature	2θ (°)	FWHM (°)	Crystallite Size (nm)
300 °C	42.92	0.85	11.18
400 °C	42.92	0.67	14.91
500 °C	42.92	0.52	17.89
600 °C	42.92	0.79	12.78
700 °C	42.92	0.91	9.95

**Table 2:** Langmuir and Freundlich isothermal adsorption models.

Langmuir				Freundlich		
K_F_/(mg/g)	K_L_/(mg/g)	R_L_	R^2^	K_F_/(mg/g)	n	R^2^
47.86	−0.996	−0.035	0.991	30.37	24.44	0.03051

**Table 3:** Langmuir and Freundlich isothermal adsorption models.

Quasi-First Order Kinetic Parameters			Quasi-Second Order Kinetic Parameters		
K_1_/min^−1^	Q_e_/(mg/g)	R^2^	K_2_/(g/mg·min^−1^)	Q_e_/(mg/g)	R^2^
−0.00645	28.37	0.24358	0.1296	45.36	0.9846

## Data Availability

The data that support the findings of this study are available from the corresponding author, F. Xia, upon reasonable request.
